# Supplementary material for: Enhancing integrated analysis of national and global goal pursuit by endogenizing economic productivity
Source: PLoS One. 2021 Feb 25;16(2):e0246797. doi: 10.1371/journal.pone.0246797 (PMC7906344; doi:10.1371/journal.pone.0246797)
Supplement: S1 Appendix — (DOCX) [file pone.0246797.s001.docx]

# S1 Appendix: Understanding and measuring TFP

Although much or most empirical analysis of economic growth has regressed possible drivers against cross-country growth rates, a lesser volume of work has differentiated the components of growth as defined in growth accounting [1] and given attention specifically to productivity. Doing so requires its conceptualization and then its measurement.

## A1. Basic conceptualization

Growth accounting using the Cobb-Douglas production function decomposes the growth rate of the economy (typically measured as the GDP growth rate) into contributions from capital, labor, and productivity, identified as either total factor productivity (TFP) or multifactor productivity (MFP) [2]. The increase in productivity is generally computed as the “Solow residual”. The calculation of the Solow residual is routinely explained in economic development texts [3: 77-85]. See also a useful summary at <https://www.ssc.wisc.edu/~ekelly/econ302/chapter8growthaccounting.pdf>. It is also possible to move some productivity enhancement change including education attainment and/or its quality into the labor term. Rather than doing that, this study built human capital, social capital, physical capital of societies (notably infrastructure), and knowledge generating capital terms and integrated them into a combined endogenous driver set for TFP. The approach follows Mankiw, *et al.* [4]. Durlauf, *et al.* [5: 578] and many others who have similarly identified human capital separately from capital and labor.

In the Cobb-Douglas function the parameter α determines the capital contribution and, with an assumption of constant returns to scale, 1-* *α represents the labor contribution.

$${GDP}_{r}={TFP}_{r}*{{(K}_{r})}^{\alpha}*{(L_{r})}^{(1-\alpha)}$$

where,

K is the capital stock in the economy

L is the labor input in the economy, often measured in million hours calculated as the labor force multiplied by average number of hours worked in the year by one laborer

α is the contribution of capital to the growth. Alpha varies across time, but in our use of this function we have used the average value of α for individual countries across time

*r* represents geographic or sociopolitical region (countries in our analysis)

To derive the Solow residual from the above formulation and to simplify the decomposition of change in the formula, we log both sides of it.

$${Log(GDP}_{r})={Log(TFP}_{r})+\left( Log\left( K_{r} \right)*\alpha\right)+(Log\left( L_{r} \right)*\left( 1-\alpha\right))$$

Taking the difference in this equation across two years and rearranging produces a form using annual change (delta).

$$\Delta Log({TFP}_{r})=\Delta Log{(GDP}_{r})-(\Delta Log\left( K)*\alpha\right)-(\Delta Log(L)*\left( 1-\alpha\right))$$

The ratio of values in each term across adjacent years (our temporal unit of analysis with most data on countries) is seldom very different from 1, for which the natural logarithm is 0. So, it is very nearly true (a small epsilon, ɛ) that the growth rates in each underlying term can be substituted for the change in the logged values.

${TFPGr}_{r}={GDPGr}_{r}-\left( KTerm*\alpha\right)-(LTerm*\left( 1-\alpha\right))$ + ɛ

where,

TFPGr is the change in TFP

GDPGr is the change in GDP

KTerm is the percentage annual change in capital stock (K) which is equal to $\Delta Log(K)$

Lterm is the percentage annual change in the labor stock (L) which is equal to $\Delta Log(L)$

This project has drawn upon data on growth, capital, labor, and productivity from the databases maintained by the Penn World Tables (PWT) and the Conference Board (CB), and upon a wide set of additional databases for the extended driver set of the TFP term. See the Penn World Table Database at <https://www.rug.nl/ggdc/productivity/pwt/> and the Conference Board Database at <https://www.conference-board.org/>.

## A2. Exploring TFP historically

The PWT and CB projects, both computing TFP historically as a residual in the growth accounting process, sometimes produce quite different results. One reason lies in the distinction between conceptualizing GDP (1) on the expenditure side (GDP^e^) and (2) on the income or what Feenstra, et al. [6], explaining the approach of the PWT, call the output side (GDP^o^). See also Box A1. At any given point in time with the prices of that time, it does not matter whether we measure GDP as the sum of private consumption, government expenditure, investment, and net exports or as the sum of value added across sector; the sums are definitionally the same within extended input-output and social accounting matrix structures (and differ from Gross National Income including a variety of international flows).

The expenditure side approach is the standard approach to looking at GDP across time, it is imbedded in most published series of GDP, and it is the source of GDP that the CB uses to compute the residual from capital and labor contributions, thereby producing large swings in TFP for some country-years, as Box A1 explains. The PWT has switched to a focus on the income (or output) side and uses price adjustment that controls the increase in the value added of the petroleum sector with its trade price. We use the output side values in subsequent analysis of productivity of PWT values and will more heavily rely on PWT than on the CB.

Box A1: GDP on the Expenditure and Output Sides, the Issue of Prices

It becomes complicated when we look across time and hold prices constant to obtain real values of GDP as a basis for computing change of the productivity residual. Consider the year-to-year change of GDP^e^ and GDP^o^ for a country like Saudi Arabia, heavily dependent on oil production and exports and with considerable state ownership. Were the price of oil to double year to year but production to remain fairly constant, the quantity produced multiplied by constant prices over time would show little change in the output side of GDP (GDP^o^) and the relatively stable productivity of the capital and labor producing that output. Yet there would be a huge jump in funds accruing to producers, especially the owners of capital. On the expenditure side, for instance from government coffers, there could be much more money chasing goods, the prices of which are likely to have changed much less on average than that of oil. If one multiplied the quantity of education, health, cars, houses, travel, etc. purchased by constant prices of them over time, it would appear that the GDP (GDP^e^) had risen substantially and, given little change in capital and labor, that productivity had surged. In fact, this is essentially what happened in Saudi Arabia with the oil price shocks of the 1970s and 1980s. With its now heavy dependence on natural gas production and exports, Qatar has similarly exhibited some separation of changes in output and expenditure side of GDP in recent years, but with a reversal of the difference shown by Saudi Arabia in the 1970s because of falling gas prices.

Although the decomposition of annual growth produces a value for the annual change of productivity, we also have an interest in its accumulated stock over time. The basic stock representation of TFP in the PWT database is as a ratio of the values in countries around the world relative to the value in the U.S., and those provide some insights into temporal dynamics (see Figure A1). Interestingly, they show a bubble in high-income countries in the 1970s and a pattern of divergence of most countries from the U.S since about 1980 until about the year 2000. After that they show a relatively constant ratio of TFP to that of the US.

Figure A1. Ratio of TFP in World Bank country economic groups to that of the United States

*Note: Values of income groups are simple averages of country members.*

*Source: IFs Version 7.61, using values from the Penn World Tables, Release 9.0*.

Figure A2 drills down into several countries on the ratios of TFP with the United States and illustrates the variation in patterns. One insight from the figure is the very slow and relative minor convergence to the U.S. levels over a long period of time by China and India (in contrast to that of South Korea). It has long been pointed out that a very significant portion of China’s dramatic growth is a function of capital stock increase (as well a movement of labor into industry) rather than productivity convergence (e.g. Krugman[7]).

 Figure A2. Ratio of TFP in selected countries to that of the United States

*Source: IFs Version 7.61, using data from the Penn World Tables, release 9.0.*

Although the ratios with U.S. productivity level are useful, they reflect changes in both the U.S. and other countries, complicating statistical analysis of TFP drivers. This project therefore used the PWT capital, labor, and GDP data (price-stabilized output side) to compute TFP for all countries. (Refer to Box A1 for summary explanation of the TFP stock series.) In doing that we needed to address another issue that complicates the exploration of drivers of productivity: the residual TFP variable is also responsive to changing exponents (alpha and beta) on capital and labor in the Cobb-Douglas function because of temporal variation in the share of value added that holders of each factor acquire. Year-to-year fluctuations in shares can be significant, for instance across the business cycle. Using variable alpha and beta across time allows a more accurate estimate of the annual residual of GDP not explained by changing magnitudes of capital stock and hours worked (laborers time average hours per year). But such fluctuations in the value of TFP introduce noise into longitudinal analysis of drivers of long-term change in productivity. Therefore, in the computation of absolute, country-specific values, we used fixed alpha and beta values for each country across our historical data (the average alpha across all years for each country individually, with beta computed as 1- alpha). İmrohoroğlu and Üngör [8] address the issue of country how specifications of alpha can produce unlikely estimations of TFP such as that of Zimbabwe exceeding that of the U.S. and suggest that it may be lower in developing countries than suggested by the PWT.

Another complication in computing country values was that the PWT did not include average hours worked for all countries, especially low-income ones. To represent the labor component of the production function consistently as the product of worker numbers and average hours worked, we used the global average of hours worked for countries without such data in the PWT.

Figure A3 illustrates results of movement to absolute country-specific values. It shows values for the same countries as in Figure A2, freeing them from the relationship to the value in the U.S. The graphic also facilities seeing magnitude of change over time, and we can see that in very general terms productivity roughly doubled in all illustrative countries across the 54-year period. In the case of the U.S., the values rose from 4.53 to 8.16, an annual rate of 1.1%. In South Korea it was 2.3%, in China it was 1.3%, and in India it was 1.7% (not suffering a Great Leap Forward immediately after 1960 as China did), indicating the potential of countries to catch up with system leaders.

 Figure A3. The stock of TFP in selected countries

*Note: The stock was computed by dividing GDP by the product of capital raised to the long-term average value of alpha (capital share of value added) and labor to 1 minus alpha.*

*Source: IFs Version 7.61, using data from the Penn World Tables, release 9.0.*

Figure A4 places change of TFP into a broader analysis of the factors underlying GDP growth. In contrast to the relatively smooth behavior of labor and even capital, the swing in productivity is perhaps surprisingly large, especially its long movement into negative territory. The large downward global swing in the Solow Residual (TFP) of Figure A4 exaggerates the reality of change in productivity potential, because part of the drop in GDP growth and therefore of this residual series reflects a decline in the utilization rate of capital and/or labor with the newly much higher prices of oil and natural gas and related financial flow and national debt challenges of the 1970s and 1980s.

Figure A4. Global GDP growth rates, with TFP, labor and capital contributions.

*Note: Data from the Conference Board produce the same pattern overall, although TFP is just above 1 percent in the 1960s and early 1970s. TFP change in the figure is the Solow Residual. All rates are 10-year moving averages.*

*Source: IFs Version 7.61, using GDP, labor, and capital from the Penn World Tables, calculating capital shares, labor shares, and TFP by authors.*

## References

1. Durlauf, S. N., Kourtellos, A. and Tan, C. M. (2008) Are Any Growth Theories Robust? *The Economic Journal* 118 (527): 29-346.
2. Zelenyuk, V. (2014 December). Testing Significance of Contributions in Growth Accounting, with Application to Testing ICT Impact on Labor Productivity of Developed Countries, *International Journal of Business and Economics*, College of Business and College of Finance, Feng Chia University, Taichung, Taiwan, vol. 13(2): 115-126.
3. Acemoglu, D. (2009) *Introduction to Modern Economic Growth*. Princeton: Princeton University Press.
4. Mankiw, N. G., Romer, D. and Weil, D. N. (1992) A contribution to the empirics of economic growth, *Quarterly Journal of Economics* 107 (2), 407–437. Also NBER Working Paper No. 3541. DOI: 10.3386/w3541.
5. Durlauf, S. N, Johnson, P. A and Temple, J. R. W. (2005) Growth Econometrics, *Handbook of Economic Growth, Volume* 1A; 555-678. Amsterdam: Elsevier North Holland. DOI: 10.1016/S1574-0684(05)01008-7.
6. Feenstra, R. C., Inklaar R., and Timmer, M. P. (2015) The Next Generation of the Penn World Table, *American Economic Review* 105(10): 3150–3182. doi.org/10.1257/aer.20130954. Available for download at [www.ggdc.net/pwt](http://www.ggdc.net/pwt)
7. Krugman, P. (1994) The Myth of Asia’s Miracle, *Foreign Affairs* 73 (6) Nov/Dec: 62-78.
8. İmrohoroğlu, A., and M. Üngör. (2016) “Is Zimbabwe More Productive than the United States? Some Observations from PWT 8.1” Economics Discussion Papers Series No. 1606. University of Otago. http://hdl.handle.net/10523/6620.
