# Supplementary material for: Enhancing integrated analysis of national and global goal pursuit by endogenizing economic productivity
Source: PLoS One. 2021 Feb 25;16(2):e0246797. doi: 10.1371/journal.pone.0246797 (PMC7906344; doi:10.1371/journal.pone.0246797)
Supplement: S2 Appendix — (DOCX) [file pone.0246797.s002.docx]

# S2 Appendix: Data sources for TFP driver analysis

All dependent and independent variable series have been added to the historical database of IFs and are therefore openly available there.
