# Supplementary material for: Enhancing integrated analysis of national and global goal pursuit by endogenizing economic productivity
Source: PLoS One. 2021 Feb 25;16(2):e0246797. doi: 10.1371/journal.pone.0246797 (PMC7906344; doi:10.1371/journal.pone.0246797)
Supplement: S3 Appendix — (DOCX) [file pone.0246797.s003.docx]

# S3 Appendix: Temporally-sliced cross-sectional analysis

Figure 1 of the text illustrated the relatively slow convergence of productivity of developing countries to the productivity levels of leading countries (see also Figures A1 and A3). The issue of convergence and divergence is an important one for the analysis of both productivity and growth. Despite more rapid population growth in the developing world than in high income countries like the U.S., there has been a pattern of convergence in GDP per capita, driven much more by the increase in capital than in productivity. Figure C1 shows capital (from the PWT) per capita in the US and China. Whereas in 1980 the ratio of that value was about 40 to 1, by 2014 it had declined to about 4 to 1. The ratio of total capital stock shifted from about 2 to 1 (US to China) to a greater stock in China.

 Figure C1. Capital stock per worker in the United States and China ($2011).

*Source: IFs Version 7.61, using data from the Penn World Tables, release 9.0.*

The growth accounting analyses here and in the text suggest the importance in long-term economic analysis of attention to the determinants of savings rates, including the very high rate that has characterized China in recent decades. Here, however, we continue to explore temporal patterns in TFP as we consider the best approach to analyzing the contribution of various drivers to its change.

Figure 2 of the text also showed the power of disruptive forces to dramatically affect the temporal patterns of change in productivity. For instance, an attempt to assess via time series regression the impact on productivity growth of increased years of educational attainment, a variable that increased quite steadily around the world in the 1970s and 1980s, would be greatly affected by the significant disruption of growth in those decades from the oil shocks (and food crises) of the first of those decades and the debt crises that followed in the second. In fact, that disruption of growth was so great as to conceal any positive contribution that important drivers of productivity such as educational advance might be having over the long run. Nor was that period the only disruption of significance that would affect statistical analysis. Clearly the economic slow-down after the financial crisis of 2006-07 would have similar implications for statistical analysis. Further, many disruptions affect a smaller subset of countries rather than most of the world. Any disruption that affect such a subset would also quite probably distort analysis of change in TFP with change in longer-term drivers.

The very significant variation of productivity advance over time also has implications for cross-sectional analysis. First, it suggests the utility of focusing on absolute levels of TFP rather than annual change. The main text does that. Second, when focusing on change as this supplemental material does, it suggests the value of using moving averages. Attention to change across time is also important, because it can provide some insight into how the contribution of drivers varies across time. The following four figures, with regional color coding of countries and selected countries labeled, relate a 10-year moving average of annual TFP change to the level of GDP per capita. Although most analysis in this report uses values from the Penn World Tables, these figures use those from the Conference Board because of their more extensive data base prior to 1980.

Figure C2 shows the pattern in 1970. In that year a disproportionate number of high-income countries (blue) fall above the line regressing a moving average of changes in TFP against GDP per capita at purchasing power parity. That year was near the end of the period in which many of the European countries continued to rebuild from the devastation of World War II and during which they benefited heavily from adaptation of technology often developed in the United States. Interestingly, the overall pattern of relationship with GDP per capita (at PPP) is positive, at least in part due to that phenomenon.^[[1]](#footnote-1)^ The upward slope of the line most probably also reflects the failure of many of the newly independent countries of Asia and especially Africa to have built capability for technological convergence. In fact, the intercept of the regression is below zero, reinforcing the rich-get-richer-and-the-poor-do-not logic (in fact, even sometimes the belief that it is at the expense of the poor) common in the dependency theory literature of the day [1: 57].

Figure C2: Smoothed annual percentage rate change in TFP as a function of GDP per capita at PPP (1970)

*Notes: On this and subsequent scatterplots in this section, Africa is green, Asia is orange, Europe is dark blue, Latin America is yellow, North America is light blue, and Oceania is red; using PWT data this relationship is much more nearly flat. Smoothing used 10-year moving average. R-squared is 0.1520.*

*Source: IFs Version 7.61, using values from the Conference Board (Erumban and de Vries [2]).*

Figure C3 shows a generally less positive picture for the European countries relative to the regression line in 1990. But it shows an even greater downward shift for the Latin American countries (yellow), which were still in the throes of the lost decades following their debt crises. In contrast, Asian countries (orange) look considerably better than earlier. This was the era of the Newly Industrialized Countries (NICs). Some of their enhanced performance should no doubt be attributed to having laid foundations in the form of some the endogenous variables of interest to us including performance in education, health, governance, and infrastructure. Yet some must also be associated with the forces unleashed by globalization and the expansion of trade and financial flows, including export-led development.

Figure C3: Smoothed annual percentage rate change in TFP as a function of GDP per capita at PPP (1990)

*Note: The relationship using PWT data is very similar. Smoothing used 10-year moving average. R-squared is 0.0225.*

*Source: IFs Version 7.61, using values from the Conference Board (Erumban and de Vries [2]).*

Figure C4 shows still a different pattern in 2000. Most striking is the change of slope from upward to downward, thereby suggesting a change in global system dynamics from one characterized heavily by divergence in productivity to one of convergence. The Soviet Union and communism in Central Europe had collapsed and many of the states (orange) doing especially well on TFP increase rates emerged in the aftermath.^[[2]](#footnote-2)^ The fact that they had heavily invested in human development earlier almost certainly helped, but so too did simple recovery of GDP lost in the transitional period. On this change of slope, see also Patel et al. [3] who note the emergence of what they call “unconditional convergence” in the 1990s, in contrast with the conclusion of Johnson and Papageorgiou [4] that convergence has not been unconditional and has been episodic and variable across countries and regions.

Figure C4: Smoothed annual percentage rate change in TFP as a function of GDP per capita at PPP (2000)

*Note: Using the TFP series computed from PWT data shifts the slope to this line to slightly upward. . Smoothing used 10-year moving average. R-squared is 0.0318*

*Source: IFs Version 7.61, using values from the Conference Board (Erumban and de Vries[2]).*

Figure C5 moves to 2010 and many post-communist countries in Central Asia and the Caucasus continue to be standouts. A considerable number of African countries also show values above those expected from the general relationship. In fact, the combined performance of those two sets of countries helped shift the slope of the line downward in both 2000 and 2010, consistent with the new pattern of developing country convergence to high-income ones.

Figure C5: Smoothed annual percentage rate change in TFP as a function of GDP per capita at PPP (2010)

*Note: Using values from the PWT the relationship is also downward sloped but somewhat less steep and strong. Smoothing used 10-year moving average. R-squared is 0.1119*

*Source: IFs Version 7.61, using values from the Conference Board (Erumban and de Vries [2]).*

The complexity of understanding TFP across time and countries can also be seen in Figure C6. It shows the GDP growth rates for China, the former Soviet states and the world as whole across time. Although the pattern for the world appears rather flat (but see again Figure 2 in the text with more detail in the scaling), that is not the case when we drill down. From 1980 onwards, following the economic reforms that began in the late 1970s, China had an average growth rate of 12%. In case of the former Soviet states, we see a sharp decline in GDP growth from 1985 to 1990 and then a very rapid rise in growth during the 1990s and later as those states began the re-construction of their economies.


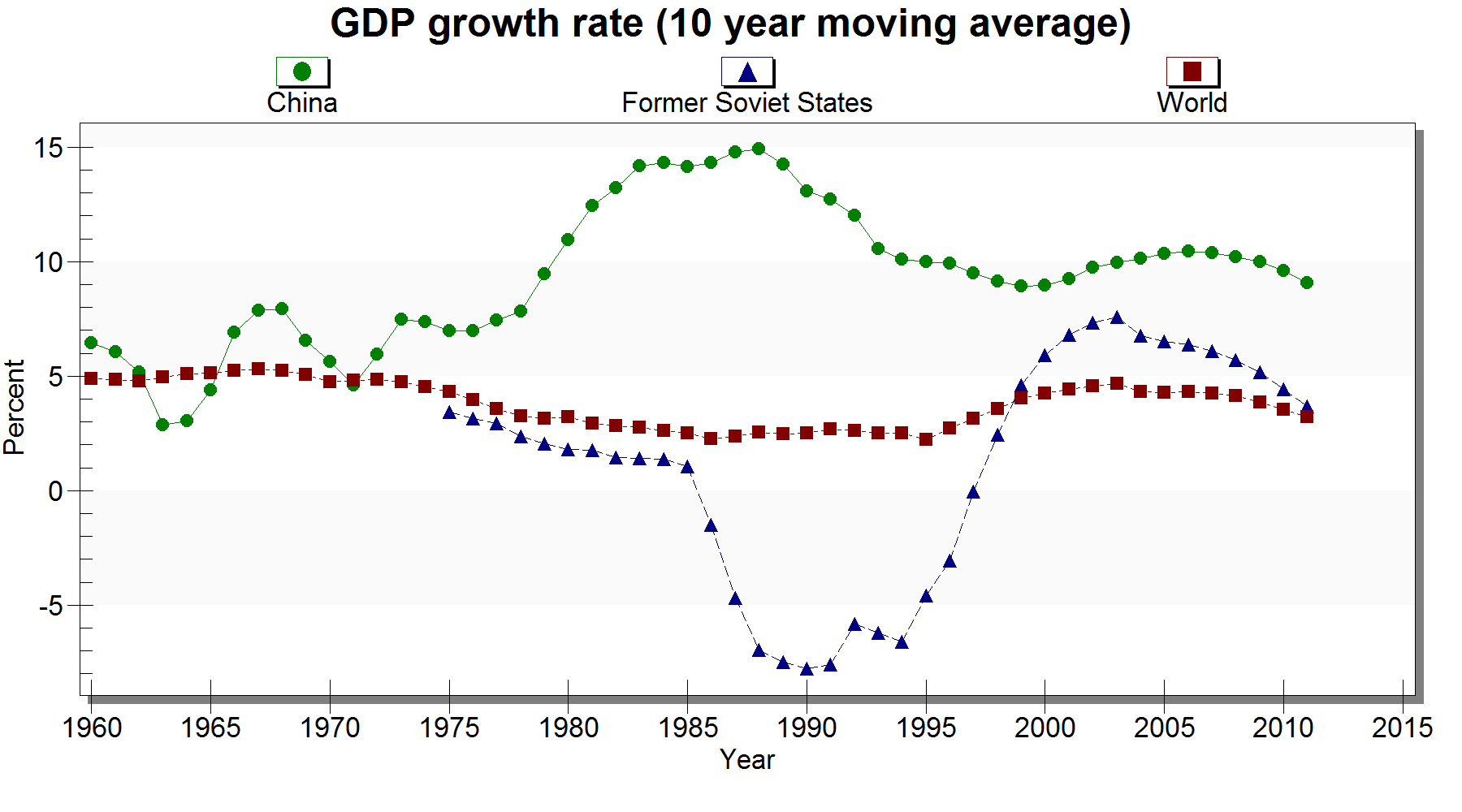


*Figure C6: Illustrative GDP growth rates: China, the FSU states, and the world*

Note: 10-year moving average.

Source: IFs Version 7.61, using values from the Conference Board.

## References

1. Wallerstein, I. (1974) *The Modern World System.* New York: Academic Press.
2. Erumban, A. A., and De Vries, K. (2016) *Global Growth Projections for the Conference Board Global Economic Outlook 2017.* Economics Program Working Paper Series EPWP #16-07. The Conference Board, New York, NY. <https://www.conference-board.org/pdf_free/workingpapers/EPWP1607.pdf>.
3. Patel, D., Sandefur, J. and Subramanian, A. (2018) *Everything You Know about Cross-Country Convergence is (Now) Wrong,* report of the Center for Global Development at <https://www.cgdev.org/blog/everything-you-know-about-cross-country-convergence-now-wrong>.
4. Johnson, P. and Papageorgiou C. (2020) What Remains of Cross-Country Convergence? *Journal of Economic Literature* 58 (1) (March) DOI: 10.1257/jel.20181207.

1. Using data from the PWT the relationship is slightly negative, but their data do not include many lower-income countries before 1990. [↑](#footnote-ref-1)
2. Data from the PWT do not show this same pattern of convergence in technology, largely because they do not include in this year many of the formerly communist countries. When they do, however, as with Armenia, the former Soviet Union’s general pattern of bulge in rates of increase in TFP is comparably visible. [↑](#footnote-ref-2)
