# Supplementary material for: Enhancing integrated analysis of national and global goal pursuit by endogenizing economic productivity
Source: PLoS One. 2021 Feb 25;16(2):e0246797. doi: 10.1371/journal.pone.0246797 (PMC7906344; doi:10.1371/journal.pone.0246797)
Supplement: S4 Appendix — (DOCX) [file pone.0246797.s004.docx]

# S4 Appendix: The International Futures (IFs) model system

The choice of integrated assessment model for this research has been the International Futures (IFs) platform. IFs has much of the desired *comprehensiveness across goal arenas*. Figure D1 shows the models in the system. Hughes [1] provided extensive elaboration of the entire system and comparison with specialized models in each issue area and with other integrated assessment models.

At the pinnacle of the IFs diagram lie the most strictly human development models, namely those for demographics (cohort-component with age-sex categories to 100+ and endogenous computations of fertility and mortality); health (15 causes of mortality and morbidity, representing or aggregating all causes of both in a model influenced by the Global Burden of disease project but linked also to Comparative Risk Assessment initiatives so as to connect drivers such as access to safe water and sanitation); and education (representing student flows across primary, lower secondary, upper secondary, and tertiary levels as well as the progression of aggregate educational attainment across the adult population as it ages).

At the supportive bottom of the figure are models with greater biophysical variable representation, including agriculture with supply and demand of food systems across crops, meat, and fish and representation of land in multiple categories; energy with oil, gas, coal, nuclear, hydroelectric and other renewable categories; and an environmental model. That environmental model represents carbon cycle flows and accumulations and generates changes in temperature and precipitation that feed to agriculture and health; it also includes a submodel of water supply and demand across renewable and effectively non-renewable (fossil water) categories. Climate change impact on productivity is represented by a combination of period-specific effects on GDP (and therefore also capital stock) and time-accumulating effects on TFP.

Between those two subsets of models, interacting with both other subsets and intermediating between them, lie those more traditionally identified as socioeconomic. The economics model is multi-sector, general equilibrium seeking over time. Production in each sector uses a Cobb-Douglas production function in which TFP, capital, and labor are all endogenously calculated with help from the other models of IFs. The model produces the total income and consumption series, as well as the Gini index for distribution, that together allow computation of the fundamentally important poverty rate indicators. The partial equilibrium physical system representations of agriculture and energy are fed into the same sectors of the general equilibrium economic model so as to enrich it and maintain accounting identities. Governance represents the dimensions of security, capacity, and inclusion with subdimensions of each. Financial accounting (governments, households, and firms) uses a social accounting matrix structure to link economics to governance, education, health, and other models. International politics represents global power, threat, and other relationships (including connections of states via foreign aid, migration, treaties and common memberships in intergovernmental organizations―connections that are far from irrelevant to the SDGs). Technology is not a truly separate model but a collection of manifestations of change across most of the other models. As such, it is also a source of human action and of interventions into more specifically human development and biophysically-linked systems. Similarly, the infrastructure model, with representations of road transport, electricity generation, water and sanitation, and information and communication technologies, is an instrument of human action that has significant impact on biophysical systems and directly serves human development (for instance, via water, sanitation, and electricity access).


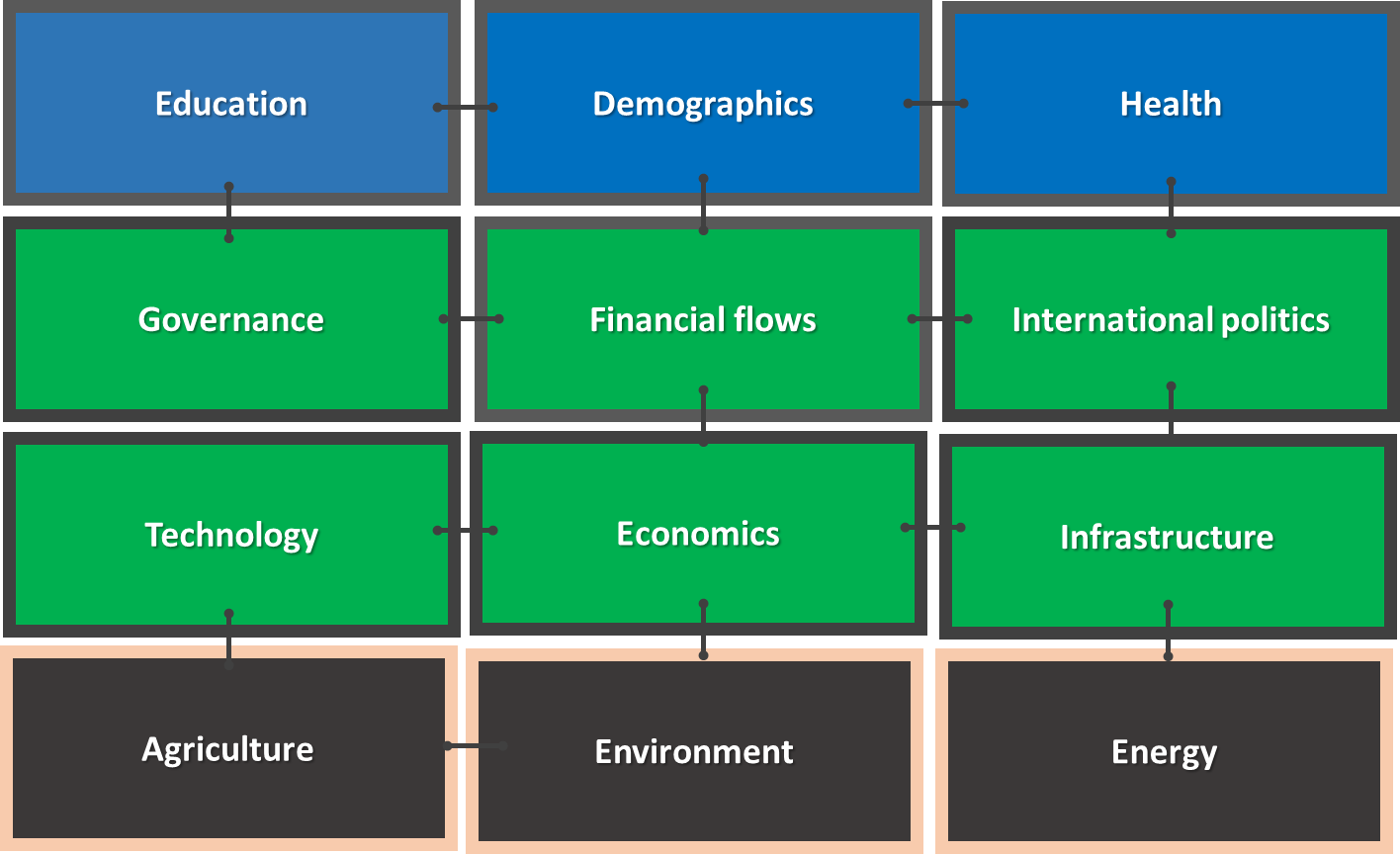


Figure D1. The models of the International Futures (IFs) system

*Note: Blue indicates models in IFs primarily focused on human development; green represents socioeconomic development; black shows models especially important to sustainable development*

*Source: Authors*

Further advantages of the IFs system include its mostly user-friendly (albeit not simple) interface, already incorporating two specialized forms on the SDGs and facilitating the use of multipliers, additive factors, and exogenous replacement of select variables as elements of scenario representation of interventions and clusters of them. Far from least, the system is open for use (in web-based and downloadable versions) and reasonably well documented at <https://pardee.du.edu/wiki/Main_Page>.

## References

1. Hughes, B. B. (2019) *International Futures:* *Building and Using Global Models.* London: Elsevier Academic Press. **ISBN:**9780128042717.
