# Supplementary material for: Enhancing integrated analysis of national and global goal pursuit by endogenizing economic productivity
Source: PLoS One. 2021 Feb 25;16(2):e0246797. doi: 10.1371/journal.pone.0246797 (PMC7906344; doi:10.1371/journal.pone.0246797)
Supplement: S5 Appendix — (DOCX) [file pone.0246797.s005.docx]

# S5 Appendix: Principal components analysis

As the text indicated, a significant issue in creating a representation of TFP that endogenizes a large number of its drivers is the multicollinearity of those drivers. Principal components analysis (PCA) facilitates extracting dimensional information from such a set of variables and was used in this project to analyze the validity of the four dimensions developed for use in the IFs system.

PCA requires a correlation matrix as an input [59]. The correlation matrix plot in Figure E1 illustrates the relationships among the drivers of productivity examined in this project (controlling for GDP per capita). PCA compresses these variables into a “loading”, or a variable weight for the resultant index.


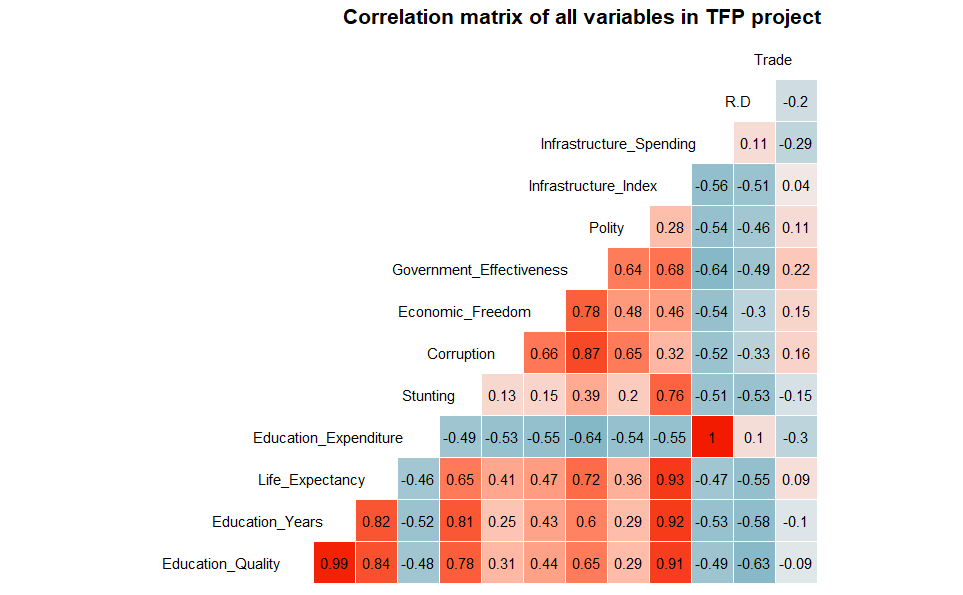


Figure E1: Correlation matrix of standardized residuals after control for GDP per capita at PPP*.*

*Note: The residuals were standardized by using z scores.*

*Source: Authors.*

Using the correlation matrix, PCA analysis can compute eigenvalues to examine the separate components that appear in the matrix. Values of more than 1.0 indicate a component that explains more variation in the dataset than any individual variable and the Kaiser criterion suggests a stopping rule for component inclusion of values greater than 1.0. The Scree Plot in Figure E2 shows that three components in E1 exceed 1.0 and a fourth is right at 1.0.


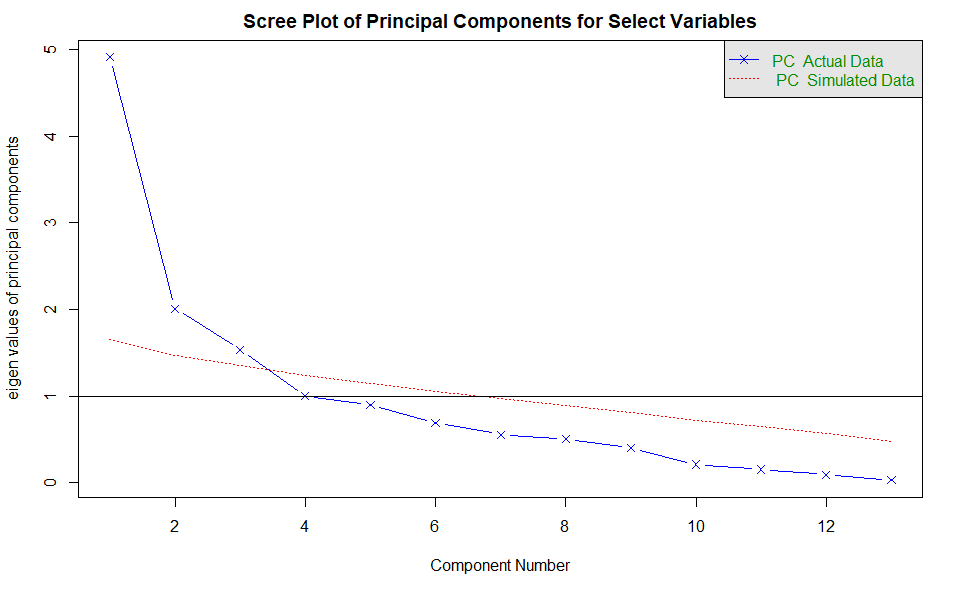


Figure E2: Scree Plot of Principal Components for Variables in Figure D1*.*

*Source: Authors.*

Figure E3 shows the principal component weights or loadings on each of the four components, including the one right at the cut-off point. The first principal component explains 28% of the variation in the dataset, the greatest share; the second component explains 22%; and the third component explains another 15%. The first component clearly clusters Human Capital terms (and also the traditional infrastructure index that we have separated out in IFs and placed with other variables in an extended Physical Capital category). The second component clearly clusters Social Capital terms.

| Variables (Components are rotated using varimax rotation) | PCA1 | PCA2 | PCA3 | PCA4 |
| --- | --- | --- | --- | --- |
| Education Quality | 0.87 | 0.23 | 0.04 | -0.11 |
| Education Years | 0.9 | 0.17 | -0.03 | -0.07 |
| Life Expectancy | 0.71 | 0.41 | 0.13 | 0.06 |
| Education Expenditure | 0.02 | 0 | 0.99 | 0.03 |
| Stunting | 0.75 | 0.01 | -0.05 | -0.08 |
| Corruption | 0.13 | 0.83 | -0.01 | -0.04 |
| Economic Freedom | 0.27 | 0.67 | 0 | 0.14 |
| Government Effectiveness | 0.44 | 0.81 | 0.01 | 0.01 |
| Polity | 0.12 | 0.67 | -0.03 | -0.28 |
| Traditional Infrastructure Index | 0.83 | 0.3 | 0.02 | 0.12 |
| Other Infrastructure Spending | 0.01 | 0.02 | 0.99 | 0.04 |
| R&D | -0.07 | 0.06 | 0.06 | 0.94 |
| Trade | 0.04 | 0.47 | 0.04 | 0.14 |

Figure E3: Correlation matrix of standardized residuals

*Source: Authors.*

The third component loads heavily only with government spending on education and infrastructure as a portion of GDP. In IFs, we have included these terns but set the weightings of them to zero with the logic that it is not spending that contributes significantly to explaining TFP, but rather the results of that spending as already represented in other variables.

The fourth component contains only research and development spending as a portion of GDP and normally would be omitted from use as a result. In the IFs system, however, that is combined also with the openness of economies (imports plus exports as a portion of GDP). Both terms relate to the original development and adaptation of knowledge/technology and therefore are closely tied to an important thrust within the theory behind understanding endogenous productivity advance.

As the main body of the report explains, the IFs representation relies on the first, second, and fourth components, creating a final index tied to extended physical capital (traditional infrastructure, ICT infrastructure, other infrastructure spending, and energy prices).

Figure E4 is a plot that visualizes the variables and the eigen values between them. See the text above for discussion of the four components that the plot helps visualize and the body of the report for the movement from the four components in Figures E3 and E4 to the operationalization in IFs.


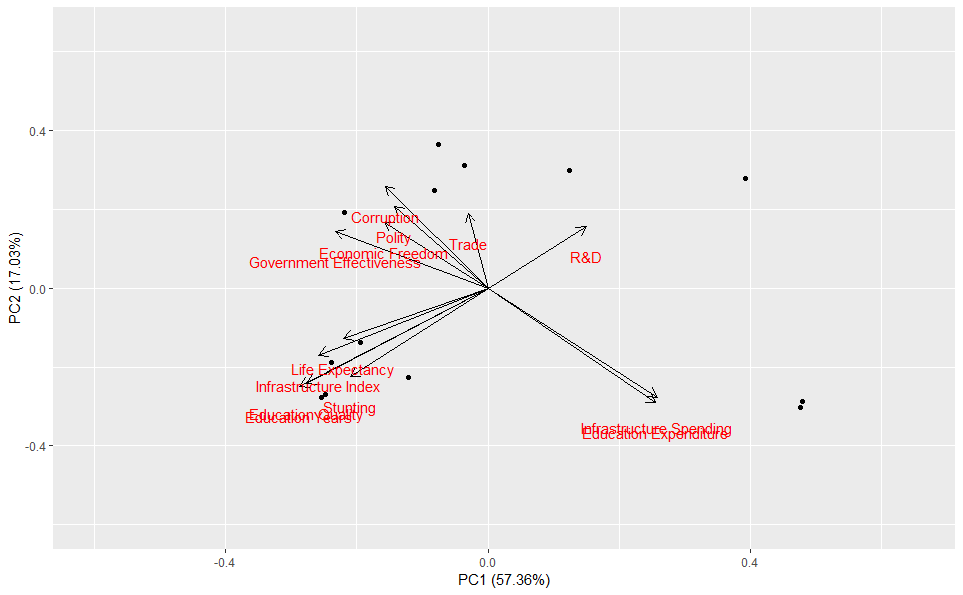


Figure E4: Plot of residual directions

*Note:* *When the PCA is run with three elements, the shares explained by the components are 28%, 22% and 18% respectively. But the screeplot above shows the shares of two components., showing 57% and 17%.*

*Source: Authors.*

## References

1. Organisation for Economic Cooperation and Development (OECD). (2008) *Handbook on Constructing Composite Indicators: Methodology and User Guide*. <https://www.oecd.org/sdd/42495745.pdf>
