# Supplementary material for: Enhancing integrated analysis of national and global goal pursuit by endogenizing economic productivity
Source: PLoS One. 2021 Feb 25;16(2):e0246797. doi: 10.1371/journal.pone.0246797 (PMC7906344; doi:10.1371/journal.pone.0246797)
Supplement: S6 Appendix — (DOCX) [file pone.0246797.s006.docx]

# **S6 Appendix: Software and data availability for use and replication**

This information builds on the general instructions for the International Futures (IFs) model system provided by the Frederick S. Pardee Center for International Futures. IFs is open source and the web-interactive version satisfies most analysis needs. The installation version runs under Windows and includes some mostly minor additional data and analysis capabilities. The software code is available under general public license and is programmed in VB6 with migration to .NET nearly complete. Data are in Access files with expected migration to SQL.

## Instructions are elaborated below for the following topics:

1. Downloading IFs and all supplementary files
2. Installing IFs
3. Running scenarios
4. Exploring results
5. Accessing supporting documentation and source code
6. Replicating analysis

## Downloading IFs

1. Explore the Pardee and IFs website at <https://pardee.du.edu/> where the web version is available for interactive use immediately.
2. For downloading, proceed to download page at <https://pardee.du.edu/access-ifs>
3. Select and download latest version.

## Installing IFs

Although IFs with its source code is open source, for replication of analysis use the packaged installation version within a Windows operating system by running IFsSetupStarter.exe. The installation may take considerable time and requires 20 GB of disk space to install and use effectively.

## Running the scenarios

The batch run feature in IFs runs multiple scenarios in succession and saves each resulting .run file. For the scenarios of this report, select the Scenario Analysis/Run/Batch Run options and sub options from the main menu bar. That will make visible all sub-folders contained within the IFs/Scenario folder of your installation. Navigate the World Integrated Scenario Sets/SDG Pathways/Synergies and Tradeoffs 2020 folder hierarchy. Then press ‘Select.’ IFs will run each scenario contained within the selected folder and save them as sperate .run files.

## Exploring results: Visualization

There are many ways in IFs to visualize variables from the Base Case and the scenarios of this project. From the Main Menu/Display option, the Flexible Displays sub-option allows a user to display a wide variety of projected variables (sometimes in combination with historical data series), create graphs, and view tables of these displays. The Flexible Displays screen also allows comparing results across countries, groups, and scenarios. Not all variables in IFs can be found in Flexible Displays; its purpose is to provide access to commonly used variables. To access the full range of variables and parameters go to Main Menu/Displays/Self-Managed Displays. For more information on using Flexible Displays, click [here](https://pardee.du.edu/wiki/Self-Managed_Display#Flexible_Display_Example) and for more information on self-managed displays, please click [here](https://pardee.du.edu/wiki/Self-Managed_Display#Display_Options_for_Self-Managed_Display).

The IFs system also includes two specialized displays for SDG goals, targets, and indicators that contain those used in this analysis and many others. Access them via Main Menu/Display/Specialized Displays for Issues/SDG Overview Table [or SDG Graph].

## Supporting documentation and the source code

IFs training manual: [Link to IFs Training Manual](https://pardee.du.edu/international-futures-ifs-training-manual)

IFs scenario guide: [Link to IFs Scenario Guide](https://pardee.du.edu/guide-scenario-analysis-international-futures-ifs)

Model documentation:

[Link to documentation files](https://pardee.du.edu/node/484)

[Link to IFs documentation Wiki](https://pardee.du.edu/wiki/Main_Page)

[Supporting data and program files at ftp site for the article](ftp://www.ifs.du.edu/CodeforTFPpaperresults.zip)

## Instructions on recreating analytical results of the paper

The material with Mendeley Data contains the following:

1. A jupyter notebook with code to re-create the results for Table 1 of this paper.
2. An R notebook to re-create the principal component analysis (PCA) results in Supplemental Appendix S5 (E).
3. The input data files for the above (which are located in the input folder).

To use these files, you will require,

1. A version of anaconda to use the jupyter notebook. This can be installed [here](https://www.anaconda.com/distribution/).
2. A version of R (Any version over 3.5) which can be downloaded [here](https://www.r-project.org/) and preferably R studio to effectively run the code which can be downloaded [here](https://rstudio.com/products/rstudio/download/).
3. All packages required are mentioned in the code which may require installation.
4. The code also mentions places where you may be required to update paths to the input data as per your requirements.

In case of questions, please contact Kanishka Narayan at [kanishkan91@gmail.com](mailto:kanishkan91@gmail.com)
