# Supplementary material for: Enhancing integrated analysis of national and global goal pursuit by endogenizing economic productivity
Source: PLoS One. 2021 Feb 25;16(2):e0246797. doi: 10.1371/journal.pone.0246797 (PMC7906344; doi:10.1371/journal.pone.0246797)
Supplement: S1 File — (DOCX) [file pone.0246797.s007.docx]

**Instructions on recreating the results mentioned in the paper**

This folder contains the following,

1. An R notebook to re-create the first 5 figures from the paper - **MFP_paper_figs.rmd**
2. A jupyter notebook with code to re-create the results for Table 1 and Table 2 mentioned in this paper on Page 15 and Page 18 respectively- **ResidualResultsforTable1&2.ipynb**
3. A R notebook to re-create the principal component analysis (PCA) results in Appendix E of the paper- **PCAAnalysis.rmd**
4. The input data files for the above (which are located in the InputData folder).

To use these files, you will require,

1. A version of anaconda to use the jupyter notebook. This can be installed [here](https://www.anaconda.com/distribution/).
2. A version of R (Any version over 3.5) which can be downloaded [here](https://www.r-project.org/) and preferably R studio to effectively run the code which can be downloaded [here](https://rstudio.com/products/rstudio/download/).
3. All packages required are mentioned in the code which may require installation.
4. The code also mentions places where you may be required to update paths to the input data as per your requirements.

In case of any questions, please contact,

Kanishka Narayan- ([kanishka.narayan@du.edu](mailto:kanishka.narayan@du.edu) or [kanishkan91@gmail.com](mailto:kanishkan91@gmail.com))

Barry Hughes- (barry.hughes@du.edu)
